# Noninvasive Biomarkers for Cardiovascular Dysfunction Programmed in Male Offspring of Adverse Pregnancy

**DOI:** 10.1161/HYPERTENSIONAHA.121.17926

**Published:** 2021-11-01

**Authors:** Rama Lakshman, Ana-Mishel Spiroski, Lauren B. McIver, Michael P. Murphy, Dino A. Giussani

**Affiliations:** Department of Physiology, Development and Neuroscience (R.L., A.-M.S., L.B.M., D.A.G.), University of Cambridge, United Kingdom.; MRC Mitochondria Biology Unit (M.P.M.), University of Cambridge, United Kingdom.; Cambridge BHF Centre of Research Excellence (A.-M.S., M.P.M., D.A.G.), University of Cambridge, United Kingdom.; Department of Medicine (M.P.M., D.A.G.), University of Cambridge, United Kingdom.; Cambridge Strategic Research Initiative in Reproduction, United Kingdom (D.A.G.).

**Keywords:** biomarkers, cardiovascular diseases, fetal hypoxia, oxidative stress, pregnancy

## Abstract

Work in preclinical animal models has established that pregnancy complicated by chronic fetal hypoxia and oxidative stress programmes cardiovascular dysfunction in adult offspring. Translating this to the human condition comes with challenges, including the early diagnosis of affected individuals to improve clinical outcomes. We hypothesize that components of programmed cardiovascular dysfunction in offspring can be identified in vivo via analysis of blood pressure variability and heart rate variability and that maternal treatment with the mitochondria-targeted antioxidant MitoQ is protective. Pregnant rats were exposed to normoxia or hypoxia (13% O_2_) ±MitoQ (500 μM in water), from 6 to 20 days gestation. Offspring were maintained in normoxia postnatally. At 16 weeks of age, 1 male per litter was instrumented with vascular catheters and a femoral blood flow probe under isoflurane anesthesia. After recovery, arterial blood pressure and femoral flow were recorded in conscious, free-moving rats and analyzed. Offspring of hypoxic pregnancy had (1) increased very-low-frequency blood pressure variability (A) and heart rate variability (B), indices consistent with impaired endothelial function and (2) increased heart rate variability low/high-frequency ratio (C) and low-frequency blood pressure variability (D), indices of cardiac and vascular sympathetic hyperreactivity, respectively. MitoQ ameliorated A and B but not C and D. We show that asymptomatic cardiovascular dysfunction in adult offspring programmed by hypoxic pregnancy can be diagnosed in vivo by blood pressure variability and heart rate variability, suggesting that these noninvasive biomarkers could be translated to the clinical setting. MitoQ protected against programmed endothelial dysfunction but not sympathetic hyperreactivity, highlighting the divergent programming mechanisms involved.

## Abstract 2

Supplemental Digital Content is available in the text.

Clinical studies in humans and data derived from preclinical rodent and ovine models have established that offspring exposed to adverse conditions in utero have an increased risk of developing cardiovascular disease in later life.^1–7^ Chronic fetal hypoxia is one of the most common outcomes of adverse human pregnancy as it can result from many complications, including preeclampsia, placental insufficiency, intrauterine infection, maternal obesity, and high-altitude pregnancy.^8^ Data show that developmental hypoxia programmes in the adult offspring impaired NO-dependent endothelial function with increased sympathetic reactivity in peripheral arterioles, as well as sympathetically dominant regulation of cardiac function.^1,6,7,9–13^ These adverse outcomes of cardiovascular dysfunction precede the development of overt disease but are strongly implicated in the pathogenesis of future hypertension, atherosclerosis, and heart failure.^14–16^ Therefore, their early diagnosis could help prevent further progression of dysfunction and the establishment of heart disease. Noninvasive diagnostics have the capacity to identify early indicators of programmed cardiovascular dysfunction in young adult offspring of complicated pregnancy.

Alterations in blood pressure variability (BPV) and heart rate variability (HRV) are clinically relevant noninvasive biomarkers suitable for human translation. Regulatory mechanisms of arterial blood pressure homeostasis include very-low-, low-, and high-frequency (VLF, LF, HF) BPV and HRV stemming from myogenic vasomotor oscillations,^17–19^ baroreflex loop resonance,^20–22^ and effects of respiration^21,23–27^ (Figure 1). A reduction in endothelial NO is known to augment myogenic vascular responses and, therefore, increase VLF BPV.^19,28–30^ Increased VLF HRV and LF BPV (baroreflex resonance) are related to enhanced sympathetic activity in the peripheral vasculature.^21–23,31,32^ LF HRV reflects the combined cardiac sympathetic and vagal inputs to the sinoatrial node, while HF HRV reflects purely cardiac vagal activity.^31,33–35^ Consequently, the LF/HF ratio of HRV is an established marker of the cardiac sympathovagal balance. Therefore, an increase in LF HRV in normalized units [LF_nu_=LF/(LF+HF)] indicates cardiac sympathetic dominance, while an increase in the HF HRV in normalized units [HF_nu_=HF/(LF+HF)] indicates vagal dominance.^31,33–35^

**Figure 1.**
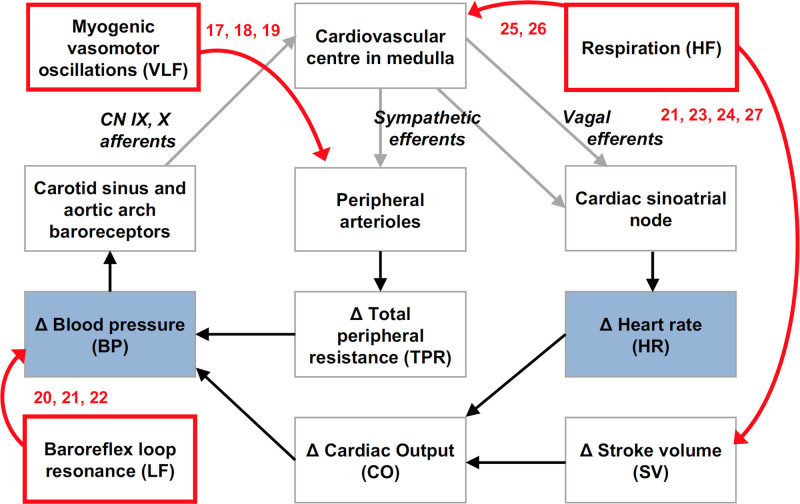
**Origins of arterial blood pressure (BP) and heart rate (HR) variability.** Acute changes in BP are restored via the baroreflex. A change in BP is detected by arterial baroreceptors, which signal to the medulla. This triggers a compensatory change in HR, and thus cardiac output [CO], via reciprocal modulation of sympathetic and vagal activity to the cardiac sinoatrial node. There is also a change in sympathetic outflow to peripheral arterioles, resulting in a compensatory change in total peripheral vascular resistance (TPR). Very-low-frequency (VLF; red box) blood pressure variability (BPV) occurs due to myogenic responses creating a VLF oscillation in peripheral arteriolar tone and thus TPR. The VLF BPV activates the baroreflex leading to compensatory VLF HRV. Low-frequency (LF) BPV and HRV originate from baroreflex loop resonance (red box). At the resonant frequency, the time delay in this negative feedback loop means the input and output are in phase, generating self-sustained oscillations. High-frequency (HF) BPV and HRV correspond to respiration (red box). The mechanical changes during respiration lead to HF BP oscillations (inspiration lowers intrathoracic pressure, leading to increased venous return, stroke volume [SV], and, therefore, CO), which then activate the baroreflex to produce compensatory HR oscillations.

Animal studies in several laboratories have established that chronic fetal hypoxia increases the generation of reactive oxygen species (ROS) in the placenta, the fetal heart, and vasculature, resulting in oxidative stress in the fetoplacenta unit.^1,3,6,7,9,36–40^ Previous work from our group in rat and sheep pregnancy reported that maternal treatment with the antioxidant vitamin C reduced fetal oxidative stress and protected against the programming of systemic hypertension in adult offspring of hypoxic pregnancy.^6,9^ However, only high doses of vitamin C, incompatible with human treatment, proved effective.^6,9^ These data indicated that a targeted antioxidant therapy providing a pharmacologically relevant dose could prove suitable for clinical translation. MitoQ is a mitochondria-targeted antioxidant, consisting of a quinone group covalently linked to a triphenylphosphonium cation by a 10-carbon chain.^41^ The cation drives MitoQ bioaccumulation within mitochondria, due to the negative transmembrane potential. Within the mitochondrial matrix, the ubiquinone group is reduced to ubiquinol by complex II of the electron transport chain, and oxidized to ubiquinone by scavenging ROS, thereby maintaining a self-sustaining pool.^41^ Because mitochondria are the main producers of cellular ROS,^42^ MitoQ is effective at low doses, providing a mitochondria-targeted therapy of improved clinical translation.^41^ Phase II trials have shown that MitoQ can be given safely to humans at doses that are protective against pathologies involving mitochondria-derived oxidative stress.^41^

Therefore, in the present study, we used an established rat model of hypoxic pregnancy to test the interrelated hypotheses that programmed cardiovascular dysfunction in the young adult offspring and its amelioration by maternal treatment with MitoQ can both be identified at the whole organism level through alterations in BPV and HRV, providing noninvasive biomarkers for clinical translation.

## Methods

The data that support the findings of this study are available from the corresponding author upon reasonable request.

### Ethical Approval

All experiments were performed under the UK Animals (Scientific Procedures) Act, 1986 Amendment Regulations 2012 following review by the University of Cambridge Animal Welfare Ethical Review Body and conducted in accordance with these regulations. Reporting conforms with the ARRIVE (Animal Research: Reporting of In Vivo Experiments) Guidelines.

### Generation of Experimental Groups

Wistar rats (Charles River, Ltd, Margate, United Kingdom) were housed under standard conditions: 21% oxygen (O_2_), 60% humidity, 21°C, and a 12-hour light/dark cycle, with free access to food (maintenance diet; Charles River, Ltd) and water. Following 14 days of acclimatization, nulliparous females were paired with fertile males (minimum 12 weeks of age). The presence of a copulatory plug was defined as day 0 of gestation. Pregnant females (dams) were housed individually under the established conditions.

**Figure 2.**
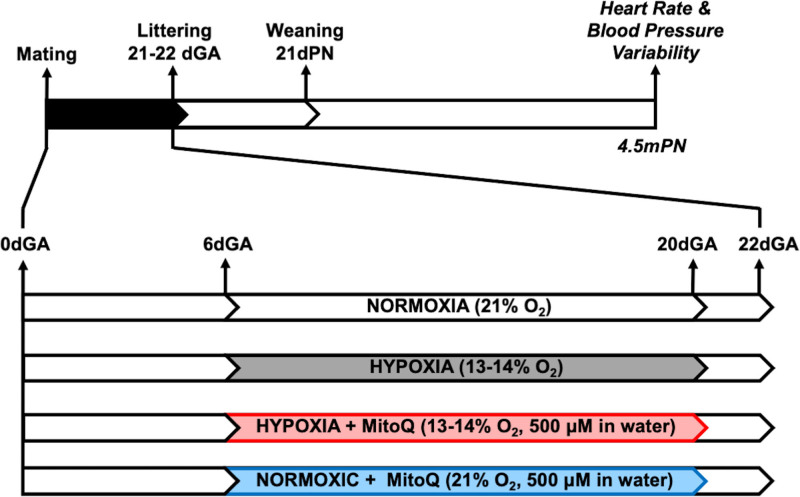
**Experimental design.** Days gestational age (dGA) for the induction of prenatal hypoxia, and MitoQ intervention, weaning at 21 postnatal days (dPN), and cardiovascular assessment at 4.5 postnatal months (mPN).

On day 6 of gestation, dams were randomly allocated to 1 of 4 groups: normoxia (N), hypoxia (H), hypoxia+MitoQ (HM), or normoxia+MitoQ (NM). From days 6 to 20 of gestation, dams in the hypoxic groups (H and HM) were placed within a chamber combining a PVC isolator and nitrogen generator. The chamber housed up to 9 rat cages in a tranquil environment.^9,43^ By varying nitrogen inflow against constant air inflow, the O_2_ fraction was maintained at 13% to 14%.^1^ This simulates the reduction in oxygenation experienced at 3500 m altitude.^44^ This level of hypoxia also results in a 20% to 30% decrease in Po_2_ in the fetal circulation,^44–46^ which corresponds to the fall in oxygenation measured by cordocentesis in human infants in pregnancy complicated by fetal growth restricted pregnancy or preeclampsia.^47,48^ Therefore, the level of hypoxic pregnancy induced is human clinically relevant. Exposing pregnant Wistar rats to 13% to 14% O_2_ from days 6 to 20 of gestation does not reduce maternal food intake,^1,9,43^ allowing the effect of hypoxia to be assessed independent of changes in maternal nutrition. Maternal hypoxia was initiated on day 6, as significant pregnancy loss can be triggered if initiated before this time point.^9,43^ From days 6 to 20 of gestation, dams in the MitoQ treatment groups (HM and NM) were provided with MitoQ at 500 μM/L in their drinking water, which was made fresh every day. Previous rodent studies show that similar doses can be given safely long term and that these doses are protective in pathological models.^1,41^ On day 20 of pregnancy, all dams were returned to normoxia and normal drinking water and allowed to litter naturally (days 21 and 22). At 2 days postnatal age, pups were sexed by measurement of anogenital distance, weighed, and litters reduced to 8 pups with an equal sex ratio to standardize feeding and maternal care. Offspring were maintained in normoxic conditions postnatally. All pups remained with their mothers until weaning at postnatal day 21. After weaning, rats were group-housed under standard conditions and maintained until 4.5 months of age (Figure 2). Maternal and offspring morphometrics were collected (Table S1 in the Supplemental Material). No rats were euthanized for the purposes of this work. Following cardiovascular assessment, all rats were allocated to undergo further experimentation published previously,^1^ after which they were euthanized by CO_2_ inhalation and posterior cervical dislocation.

### Cardiovascular Assessment In Vivo

To control for sex and within litter variation, 1 male per litter was randomly assigned for cardiovascular assessment at 4.5 months of age. Rats were surgically instrumented under 2.0% to 2.5% isoflurane general anesthesia. Adequate depth of anesthesia was confirmed and monitored by the absence of corneal and limb withdrawal reflexes. The femoral artery and vein were isolated under a dissecting microscope, and catheters prefilled with heparinized saline (100 U·mL^−1^ heparin in 0.9% NaCl) were introduced.^1,43^ A customized Transonic flow probe (0.7 PSL Back Exit NanoProbe; Transonic, United States) was implanted around the femoral artery of the contralateral leg, and the flow probe and catheters were exteriorized at the shoulder with a dual-channel vascular access harness.^1^

Rats were acclimatized to the testing cage for 30 minutes daily for 4 to 5 days and immediately before the testing procedures. On the fifth postoperative day, the arterial catheter was flushed and connected to a fluid-filled pressure transducer. Continuous baseline descending aortic arterial blood pressure and femoral blood flow were recorded for 30 minutes with a PowerLab 4/25 on LabChart Pro 8.0 (both AD Instruments). All recordings were made in the afternoon to control for the effect of circadian rhythm. Spontaneous movement was noted on the LabChart recording.^1^

### Blood Pressure and HRV Analysis

Two nonoverlapping 5-minute epochs from the 30-minute recording at the same time of the day were selected for BPV and HRV analysis. In this study, 5-minute recording epochs were selected to optimize the accuracy of VLF component analysis. Because rodents have a much higher heart rate than humans, <5-minute epochs of recording are acceptable.^49^ The 5-minute epochs were selected to be as close to the end of the basal recording period as possible, with those containing movement artifacts excluded. Each epoch was inspected manually and the peak detection height adjusted until all peaks were detected.

**Figure 3.**
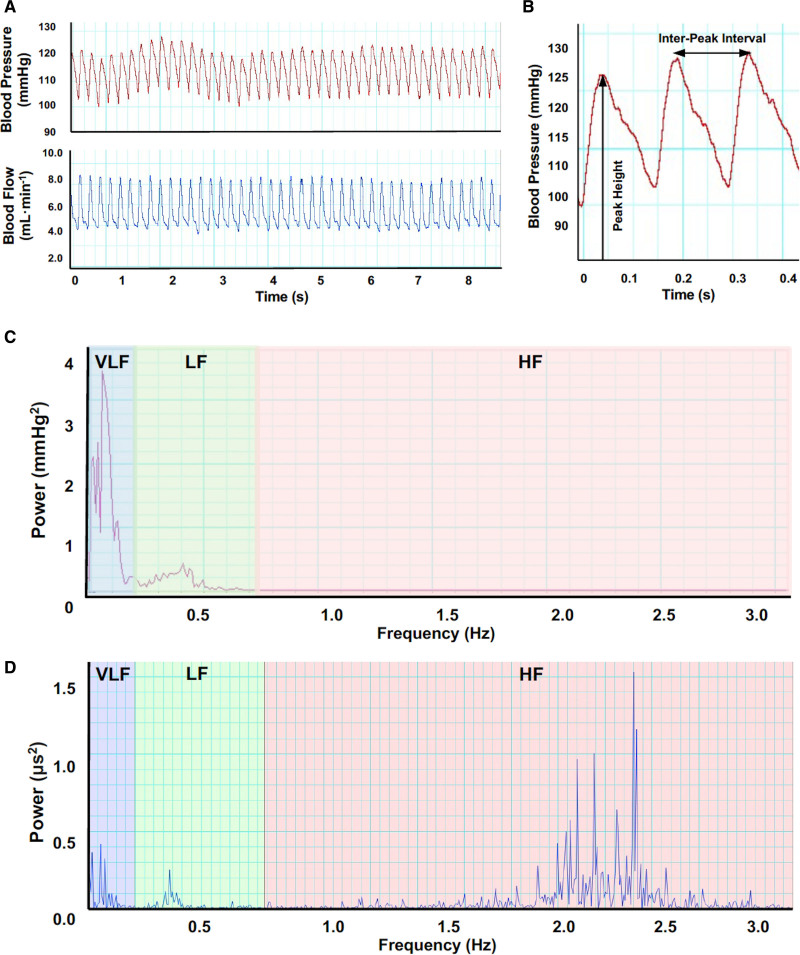
**Cardiovascular recording analysis.** Representative LabChart recording of continuous, pulsatile arterial blood pressure (red trace) and femoral blood flow (blue trace) from a normoxic rat (**A**). Expanded section of the blood pressure trace (**B**); the peak height (systolic blood pressure) and interpeak interval are noted. Values for systolic blood pressure and interpeak interval were plotted against time and fast Fourier transformed into the frequency domain to produce blood pressure variability (BPV; **C**) and heart rate variability (HRV; **D**) power spectra, respectively. HF indicates high frequency; LF, low frequency; and VLF, very low frequency.

Systolic BPV was calculated based on the variation in the peak heights in the blood pressure recording and HRV based on the variation in interpeak interval lengths in either the blood pressure or blood flow recording (Figure 3). For BPV analysis, the values for systolic blood pressure were plotted against time, resampled at 10 Hz in accordance with the Nyquist-Shannon sampling theorem,^50,51^ and transformed into the frequency domain using the Fast Fourier Transform algorithm (Fast Fourier Transform size 1 KHz, Hann window with 50% overlap). The DC component at 0 Hz was removed. For HRV analysis, the values for inter-heartbeat interval were plotted against time, and the SD of interbeat intervals—a commonly reported time domain measure of overall HRV—was calculated. The data were then fast Fourier transformed using the HRV analysis module in LabChart. For both BPV and HRV, the amount of variation at each frequency was displayed as a power spectrum (Figure 3). LabChart was programmed to calculate the power in the VLF, LF, and HF frequency ranges. For HRV, the LF/HF ratio, LF_nu_ [=LF/(LF+HF)], and HF_nu_ [=HF/(LF+HF)] were also calculated. The frequency boundaries were set at VLF, 0–0.2 Hz; LF, 0.2–0.75 Hz, and HF, 0.75–3.0 Hz based on previous studies in adult Wistar.^52^ Visual inspection of the spectra showed that each of the main peaks was confined to one frequency range, confirming that these boundaries were appropriate.

Systolic blood pressure has been used for BPV analysis in previous rodent studies.^17,53,54^ Although HRV is traditionally calculated based on the interval between R waves in an ECG, studies have shown that blood pressure and flow recordings provide comparable results.^55–57^ Some rats had pulsatile recordings for blood flow but not blood pressure possibly due to displacement of the catheter, so only HRV could be analyzed.

### Statistical Analysis

Based on previous cardiovascular studies of offspring of hypoxic pregnancy, we calculated that to detect a statistically significant difference in femoral vascular resistance in adult rats of 25%, with 95% power and a 2-tailed significance of 0.05, n=8 per experimental group was required. Allocation to treatment was randomized, and analysis was blinded to avoid bias. All graphical and statistical analyses were performed using GraphPad Prism 7 (GraphPad Software, Inc). Distribution was verified with the Shapiro-Wilk test, and statistical comparisons were made using 1-way ANOVA for differences between the groups and 2-way ANOVA for the effect of hypoxia, the effect of MitoQ, and any interaction. For correlations between measures, the Pearson correlation coefficient (R^2^) was calculated. For all comparisons, significance was set at *P*<0.05. Data are expressed as the mean±SEM.

## Results

### Basal Arterial Blood Pressure and Heart Rate

**Figure 4.**
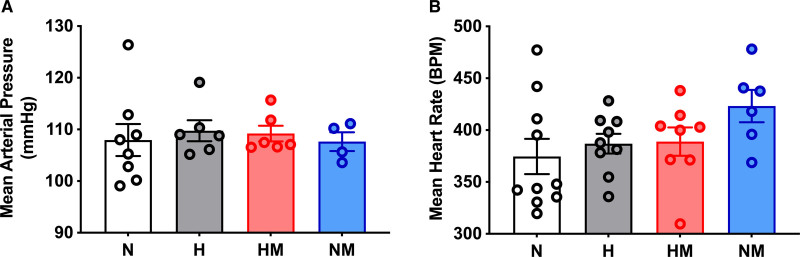
**Baseline cardiovascular function.** Mean arterial pressure (**A**) and mean heart rate (**B**) in male offspring from normoxic (N; white, n=8 and 10, respectively), hypoxic (H; gray, n=6 and 9, respectively), hypoxic+MitoQ (HM; red, n=6 and 8, respectively), and normoxic+MitoQ (NM; blue, n=4 and 6, respectively) pregnancies. Data are mean±SEM. Two-way ANOVA for the effect of hypoxia (*P<0.05) and the effect of MitoQ (†*P*<0.05). BPM indicates beats per minute.

When the offspring were 4.5 months, mean arterial blood pressure was not significantly different (*P*=0.42) between the four treatment groups (Figure 4A). Mean heart rate was also not significantly different (*P*=0.33) between the four treatment groups (Figure 4B).

### Systolic BPV

**Figure 5.**
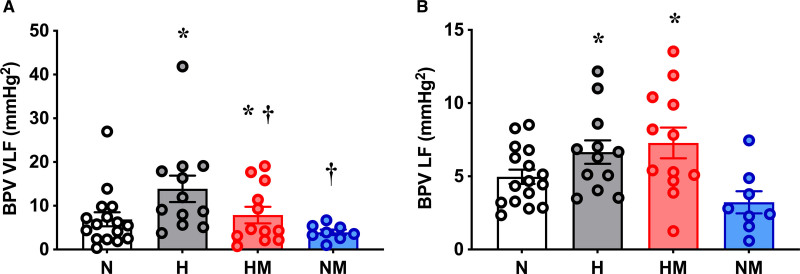
**Blood pressure variability (BPV).** Very-low-frequency (VLF; **A**) and low-frequency (LF; **B**) blood pressure variability (BPV) in male offspring from normoxic (N; white, n=8), hypoxic (H; gray, n=6), hypoxic+MitoQ (HM; red, n=6), and normoxic+MitoQ (NM; blue, n=4) pregnancies. Data are mean±SEM. Two-way ANOVA for the effect of hypoxia (*P<0.05) and the effect of MitoQ (†*P*<0.05).

Offspring of hypoxic pregnancy had increased (*P*=0.026) VLF BPV compared with normoxic offspring. Offspring from pregnancies treated with maternal MitoQ had decreased (*P*=0.049) VLF BPV compared with offspring from untreated pregnancies, and there was no interaction (*P*=0.37) between the effect of hypoxia and MitoQ (Figure 5A). Offspring from hypoxic pregnancy also had increased (*P*=0.001) LF BPV, but here, maternal MitoQ had no effect (*P*=0.49; Figure 5B). HF BPV was not different among the experimental groups (*P*=0.75: N, 0.57±0.15 mm Hg^2^; H, 0.98±0.48 mm Hg^2^; HM, 0.77±0.24 mm Hg^2^; NM, 1.0±0.58 mm Hg^2^).

### Heart Rate Variability

**Figure 6.**
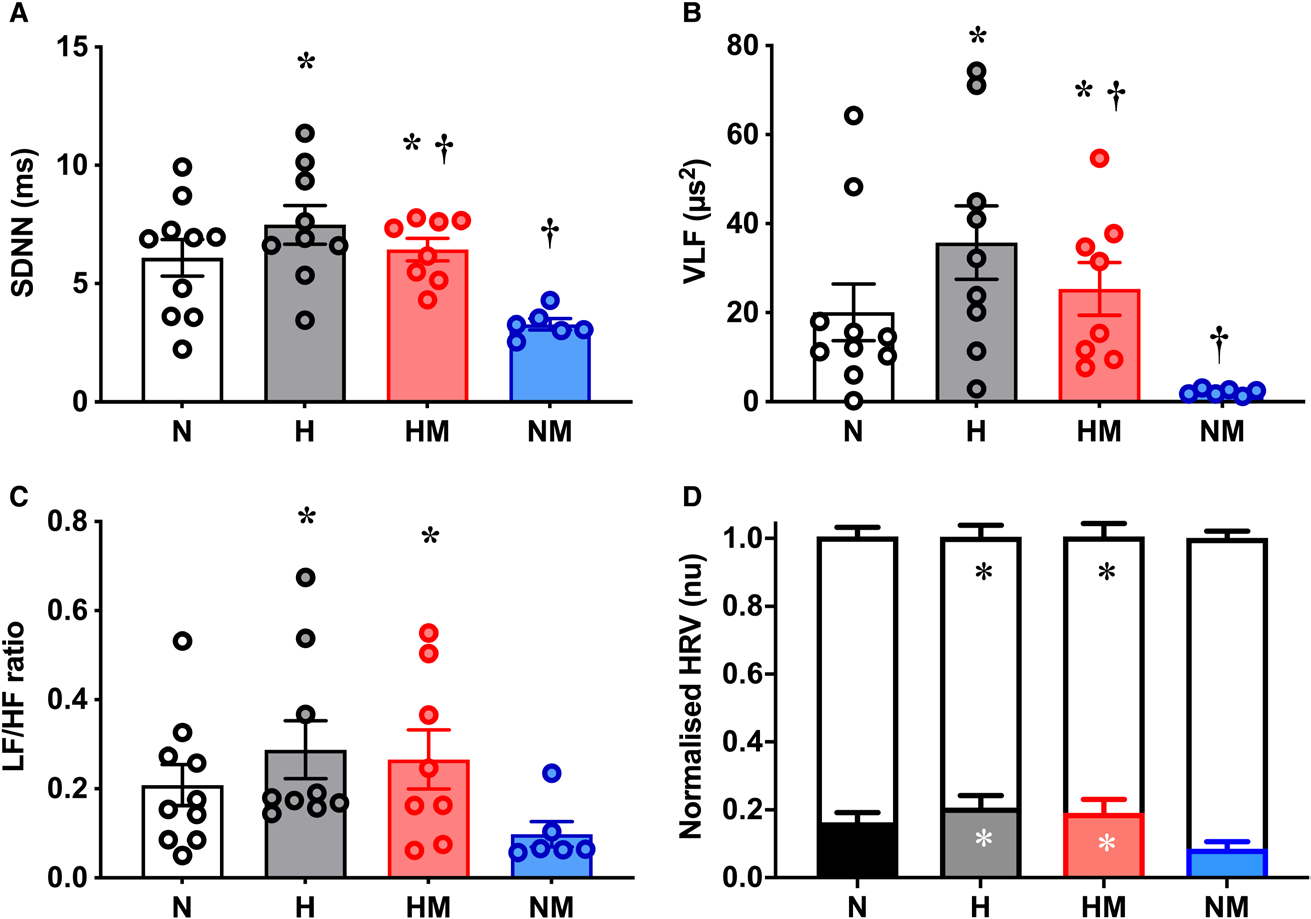
**Heart rate variability.** The SD of the interbeat intervals (SDNNs; **A**), very-low-frequency (VLF) heart rate variability (HRV; **B**), low-frequency (LF)/high-frequency (HF) ratio (**C**) in male offspring from normoxic (N; white, n=10), hypoxic (H; gray, n=9), hypoxic+MitoQ (HM; red, n=8), and normoxic+MitoQ (NM; blue, n=6) pregnancies, and normalized LF (black bars) and HF (white bars) HRV (**D**). Data are mean±SEM. Two-way ANOVA for the effect of hypoxia (*P<0.05) and the effect of MitoQ (†*P*<0.05).

Offspring of hypoxic pregnancy had increased SD of interbeat intervals HRV (*P*=0.003), while this was decreased in offspring from pregnancies treated with maternal MitoQ (*P*=0.011). There was no interaction (*P*=0.23) between hypoxia and MitoQ (Figure 6A). Similarly, offspring of hypoxic pregnancy had increased (*P*=0.008) VLF HRV, whereas those from maternal MitoQ-treated pregnancies showed decreased (*P*=0.045) VLF HRV. There was no interaction (*P*=0.58) between hypoxia and MitoQ (Figure 6B). LF HRV was not significantly different among experimental groups (*P*=0.077: N, 2.43±0.88 μs^2^; H, 2.18±0.46 μs^2^; HM, 2.99±1.08 μs^2^; NM, 0.27±0.069 μs^2^). HF HRV was also not different (*P*=0.26: N, 9.75±2.83 μs^2^; H, 8.79±2.14 μs^2^; HM, 10.43±3.18 μs^2^; NM, 2.86±0.19 μs^2^). Offspring of hypoxic pregnancy had increased (*P*=0.040) LF/HF ratio of HRV, whereas those from maternal MitoQ treatment showed no effect (*P*=0.26; Figure 6C). Offspring of hypoxic pregnancy also had increased (*P*=0.036) LF_nu_ HRV and decreased (*P*=0.034) HF_nu_ HRV, and again, those from maternal MitoQ treatment showed no effect (*P*=0.18; Figure 6D).

### Correlations

Data from all four groups showed significant positive correlations between VLF BPV and VLF HRV (Figure S1 in the Supplemental Material) and between the LF/HF ratio of HRV and LF_nu_ HRV (Figure S1 in the Supplemental Material).

## Discussion

Consistent with our hypothesis, we show that male young adult rat offspring of hypoxic pregnancy, which we have previously reported to show abnormal cardiovascular function,^1,5,8,43^ display biomarkers of impaired endothelial NO-dependent vasodilatation (increased VLF BPV and VLF HRV), as well as vascular and cardiac sympathetic hyperreactivity (increased LF BPV and LF/HF ratio HRV, respectively), before the development of overt cardiovascular disease. Importantly, these biomarkers of cardiovascular dysfunction can be identified noninvasively in humans. Maternal MitoQ therapy was protective against indices associated with impaired endothelial function but had no effect on vascular or cardiac sympathetic hyperreactivity in adult offspring of hypoxic pregnancy, highlighting the divergent programming mechanisms involved.

Alterations in BPV and HRV are clinically translatable biomarkers that can be collected from patients during routine analysis, such as with a beat-to-beat finger blood pressure monitor and ECG.^58^ Importantly, the relatively young offspring in this study, at an age that corresponds to late adolescence in humans,^59^ were not hypertensive or tachycardic and did not display symptoms of cardiovascular disease. In humans, the programmed cardiovascular changes indicated by these biomarkers precede the development of overt cardiovascular pathology. Both impaired NO-dependent vasodilatation^16^ and increased vascular sympathetic activity^14^ are implicated in the pathogenesis of hypertension. Additionally, a sustained increase in cardiac sympathetic activity stimulates cardiomyocyte apoptosis and β-adrenergic desensitization, which contribute to the pathology of heart failure.^15^ We know offspring of hypoxic pregnancy are at increased risk of these diseases in later life.^3^ Therefore, BPV and HRV could enable earlier detection of programmed cardiovascular dysfunction and intervention.

The reasoning behind using alterations in BPV and HRV as biomarkers of cardiovascular dysfunction is underpinned by the physiology of blood pressure homeostasis. A reduction in endothelial NO-dependent vasodilatation is known to increase VLF BPV by reducing NO-mediated buffering of VLF vascular myogenic contractions.^19,28–30^ VLF HRV is proposed to be a compensatory baroreflex response to VLF BP oscillations.^23^ Accordingly, in the present study, we found VLF BPV and HRV to be positively correlated (R^2^=0.58). Baroreflex loop resonance generates an LF oscillation in sympathetic outflow to the peripheral vasculature, resulting in LF vasoconstriction.^20,22,60^ As endothelial mediators cannot act fast enough to buffer vasoconstriction at this frequency, LF BPV is an established biomarker of vascular sympathetic activity.^21,22,31,32^ LF HRV also corresponds to the baroreflex resonant frequency and represents combined sympathetic and parasympathetic cardiac modulation, while HF HRV, coupled with the respiratory frequency, represents purely the faster acting parasympathetic modulation.^49^ Therefore, the LF/HF ratio of HRV and LF_nu_ are established measures of cardiac sympathovagal balance.^31,33–35^ In the present study, the robust correlation (R^2^=0.99) between the LF/HF ratio and LF_nu_ HRV demonstrates their equivalence.

Additional data in the present study show that maternal MitoQ treatment in hypoxic pregnancy protected against the programming of indices associated with impaired endothelial NO-dependent vasodilatation, suggesting that this programming is mediated developmentally by mitochondria-derived oxidative stress or redox signaling. Importantly, the protection afforded by MitoQ treatment in hypoxic pregnancy could also be identified through noninvasive analysis of VLF BPV and HRV. One proposed mechanism underlying the programming of cardiovascular dysfunction in offspring of hypoxic pregnancy is that fetal hypoxia results in increased mitochondrial production of the ROS superoxide (O_2_•^−^), which rapidly reacts with endothelial-derived NO, reducing its bioavailability.^3,61^ By scavenging excess mitochondria-derived O_2_•^−^ production, or by decreasing its production, MitoQ can restore NO bioavailability and thereby endothelial function. This is supported by work in hypoxic sheep pregnancy, where maternal MitoQ treatment has been found to protect against the programming of hypertension in adulthood by enhancing NO signaling in the peripheral vasculature.^7^ It is also consistent with work describing that hypoxic incubation of chicken embryos can enhance mitochondria-derived ROS production and that this is prevented by MitoQ treatment.^7^ It is also consistent with work in adult spontaneously hypertensive rats, which showed that MitoQ increases NO bioavailability and improves endothelial function.^62^ The protective effects of MitoQ on the developing cardiovascular system may be direct or secondary to beneficial effects at the level of the placenta. Using a mitochondria-targeted mass spectrometry probe, we have previously reported that incubation of chicken embryos under hypoxic conditions increases the generation of mitochondria-derived ROS.^7^ The same study showed that treatment of hypoxic embryos with MitoQ normalizes mitochondria-derived ROS generation, confirming a direct protective effect of MitoQ on the embryonic cardiovascular system. Similarly, we and others have reported that maternal treatment with both authentic MitoQ and nanoparticle bound MitoQ in hypoxic pregnancy reduces oxidative stress and has protective effects on the maternal side of the placenta.^37,38,40,63^

Conversely, the present study shows that maternal MitoQ therapy had no significant effect on indices associated with either vascular (LF BPV) or cardiac (LF/HF ratio and LF_nu_ HRV) sympathetic hyperreactivity. These findings suggest that the programming of sympathetic hyperreactivity in offspring of hypoxic pregnancy is mediated via mechanisms independent of mitochondria-derived oxidative stress. Fetal hypoxia is known to activate a carotid chemoreflex, which increases sympathetic outflow, mediating vasoconstriction in the peripheral vasculature.^8,64^ This effect is part of the fetal brain-sparing response that shunts blood flow away from less essential vascular beds toward the fetal brain.^8,64^ Persistent chemoreflex activation has been shown to lead to chemoreflex sensitization.^65^ Additionally, there is evidence that chronic developmental sympathetic stimulation can lead to upregulation of adrenoceptors and a sustained increase in tissue sensitivity.^66^ Studies in chicken embryos have reported that developmental hypoxia programmes sympathetic hyperinnervation of the peripheral vasculature that persists into adulthood; the proposed mechanism involves activation of hypoxia-inducible factor.^12,67^ Hypoxic pregnancy in rats leads to increased femoral vasoconstrictor responses to sympathetic agonists in newborn pups,^13^ as well as increased muscle sympathetic nerve activity and sympathetic hyperinnervation in adult offspring.^68^ Similarly, chronic hypoxia in ovine pregnancy enhances femoral vasoconstrictor responses to the α-adrenergic agonist phenylephrine in the fetus and programmes femoral vasoconstrictor hyperreactivity to sympathetic agonists in the adult offspring.^6^ Combined, these data suggest that chronic hypoxia programmes cardiovascular dysfunction in the adult offspring via multiple mechanisms, including mitochondria-derived oxidative stress and hyperreactivity of the sympathetic nervous system. Therefore, maternal treatment with MitoQ in hypoxic pregnancy protected against cardiovascular symptoms triggered by mitochondria-derived oxidative stress but not via enhanced sympathetic activation. Importantly, this can be identified by differential diagnoses of the noninvasive biomarkers.

These data are of clinical relevance as they highlight that pharmacological targeting of one oxidative stress pathway may be insufficient to protect offspring from cardiovascular dysfunction programmed developmentally by adverse intrauterine conditions in human complicated pregnancy. These data also highlight that noninvasive BPV and HRV monitoring in young adult offspring of complicated pregnancy can both identify clinically relevant indicators of cardiovascular dysfunction and differentiate clinical indicators mediated via oxidative stress or sympathetic hyperreactivity. Therefore, noninvasive differential diagnosis could refine early intervention with mechanism-targeted therapies in young adult offspring, before the establishment of over cardiovascular disease.

Previous studies of developmental programming in rodent models have identified sex differences in outcomes in adult offspring^69^. A limitation of this study is that by investigating male but not female offspring, sex differences were controlled for, but not addressed. An important advantage of BPV and HRV analyses presented in this study is that these measures can be conducted repeatedly across time. Therefore, follow-up work should determine longitudinal changes in BPV and HRV function over time with aging, in both male and female offspring.

In summary, using an established rat model of hypoxic pregnancy, this study shows that known components of programmed cardiovascular dysfunction can be identified in vivo in asymptomatic male adult offspring using alterations in BPV and HRV biomarkers. Our findings also provide evidence that maternal treatment with the mitochondria-targeted antioxidant MitoQ in hypoxic pregnancy protects against the programming of indices associated with reduced NO-dependent vasodilatation but not vascular or cardiac sympathetic hyperreactivity. This suggests that mitochondria-derived oxidative stress is one of the multiple mechanisms mediating cardiovascular programming by chronic fetal hypoxia. Clinically translatable findings include the use of BPV and HRV analysis for early identification of programmed cardiovascular dysfunction in human offspring of hypoxic pregnancy, as well as for diagnosis of effective intervention.

## Perspectives

Humans exposed to adverse conditions in utero have an increased cardiovascular risk in later life. Chronic fetal hypoxia is one of the most common adverse conditions in complicated pregnancy, and it is known to programme endothelial dysfunction and sympathetic hyperreactivity in preclinical animal models. Here, we report in male rats that corresponding alterations in BPV and HRV can be detected in vivo in young adult offspring of hypoxic pregnancy, before the development of overt heart disease. Therefore, BPV and HRV analysis could be useful noninvasive biomarkers for early identification of subclinical programmed cardiovascular dysfunction in humans. We also show that maternal treatment with the mitochondria-targeted MitoQ in hypoxic pregnancy prevents the programming of indices associated with endothelial dysfunction, but not of sympathetic hyperreactivity, in the adult offspring. Therefore, programmed cardiovascular disease and underlying mechanisms can be differentially diagnosed using biomarkers that can be measured noninvasively in the human clinical setting. This perspective offers the improved clinical diagnosis and targeted treatment of offspring affected by cardiovascular dysfunction, which has been programmed by their own adverse intrauterine environment.

## Article Information

### Acknowledgments

We would like to thank the staff at the Cambridge University Biomedical Services for their excellence in animal care and the Giussani Lab members for their assistance in this work. A.-M. Spiroski, D.A. Giussani, R. Lakshman, and M.P. Murphy conceived and designed the experiments. A.-M. Spiroski, D.A. Giussani, R. Lakshman, and L.B. McIver acquired, analyzed, and interpreted the data. R. Lakshman, A.-M. Spiroski, M.P. Murphy, and D.A. Giussani drafted the manuscript. A.-M. Spiroski, R. Lakshman, L.B. McIver, M.P. Murphy, and D.A. Giussani revised the manuscript, contributed to discussion, and approved the final version.

### Sources of Funding

This work was supported by the British Heart Foundation (Giussani Laboratory: PG/14/5/30547). Work in the Murphy Laboratory is supported by the Medical Research Council UK (MC_U105663142) and by a Wellcome Trust Investigator Award (110159/A/15/Z).

### Disclosures

M.P. Murphy consults for MitoQ, Inc. The other authors report no conflicts.

## Supplementary Material



## Novelty and Significance

### What Is New?

- Here, we show in rats that cardiovascular problems in male adult offspring programmed by hypoxic pregnancy can be diagnosed in vivo simply by analysis of blood pressure and heart rate variability, identifying noninvasive biomarkers that could be translated to the human clinical setting.

### What Is Relevant?

- Pregnancy affected by chronic fetal hypoxia, one of the most common pregnancy complications in humans, increases the risk of cardiovascular dysfunction in later life, including hypertension.

### Summary

We have identified noninvasive clinical biomarkers to pick up asymptomatic cardiovascular dysfunction in male adult offspring programmed by developmental hypoxia.
