# Non-Invasive Biomarkers for Cardiovascular Dysfunction Programmed in Male Offspring of Adverse Pregnancy

**DOI:** 10.1161/HYPERTENSIONAHA.121.17926

**Published:** 2021-11-01

**Authors:** Rama Lakshman, Ana-Mishel Spiroski, Lauren B. Mclver, Michael P. Murphy, Dino A. Giussani

**Affiliations:** 1 Department of Physiology, Development and Neuroscience, University of Cambridge, UK; 2 MRC Mitochondria Biology Unit, University of Cambridge, UK; 3 Cambridge BHF Centre of Research Excellence, University of Cambridge, UK; 4 Department of Medicine, University of Cambridge, UK; 5 Cambridge Strategic Research Initiative in Reproduction, Cambridge, UK

**Keywords:** biomarker, fetal hypoxia, cardiovascular disease, pregnancy, developmental programming, oxidative stress, mitochondria-targeted therapy

## Abstract

Work in pre-clinical animal models has established that pregnancy complicated by chronic fetal hypoxia and oxidative stress programmes cardiovascular dysfunction in adult offspring. Translating this to the human condition comes with challenges, including the early diagnosis of affected individuals to improve clinical outcomes. We hypothesise that components of programmed cardiovascular dysfunction in offspring can be identified *in vivo* via analysis of blood pressure variability (BPV) and heart rate variability (HRV), and that maternal treatment with the mitochondria-targeted antioxidant MitoQ is protective. Pregnant rats were exposed to normoxia or hypoxia (13% O_2_) ± MitoQ (500μM in water), from 6-20 days gestation. Offspring were maintained in normoxia postnatally. At 16 weeks of age, one male per litter was instrumented with vascular catheters and a femoral blood flow probe under isoflurane anaesthesia. After recovery, arterial blood pressure and femoral flow were recorded in conscious, free-moving rats, and analysed. Offspring of hypoxic pregnancy had: 1) increased very low frequency (VLF) BPV (A) and HRV (B), indices consistent with impaired endothelial function; and 2) increased HRV low frequency (LF)/high frequency (HF) ratio (C) and LF BPV (D), indices of cardiac and vascular sympathetic hyper-reactivity, respectively. MitoQ ameliorated A and B, but not C and D. We show that asymptomatic cardiovascular dysfunction in adult offspring programmed by hypoxic pregnancy can be diagnosed *in vivo* by BPV and HRV, suggesting that these non-invasive biomarkers could be translated to the clinical setting. MitoQ protected against programmed endothelial dysfunction but not sympathetic hyper-reactivity, highlighting the divergent programming mechanisms involved.

## Introduction

Clinical studies in humans, and data derived from preclinical rodent and ovine models have established that offspring exposed to adverse-conditions *in utero* have an increased risk of developing cardiovascular disease in later life.^1–7^ Chronic fetal hypoxia is one of the most common outcomes of adverse human pregnancy, as it can result from many complications, including preeclampsia, placental insufficiency, intrauterine infection, maternal obesity and high altitude pregnancy.^8^ Data show that developmental hypoxia programmes in the adult offspring impaired NO-dependent endothelial function with increased sympathetic reactivity in peripheral arterioles, as well as sympathetically dominant regulation of cardiac function.^1, 6, 7, 9–13^ These adverse outcomes of cardiovascular dysfunction precede the development of overt disease, but are strongly implicated in the pathogenesis of future hypertension, atherosclerosis and heart failure.^14–16^ Therefore, their early diagnosis could help prevent further progression of dysfunction and the establishment of heart disease. Non-invasive diagnostics have the capacity to identify early indicators of programmed cardiovascular dysfunction in young adult offspring of complicated pregnancy.

**Figure 1:**
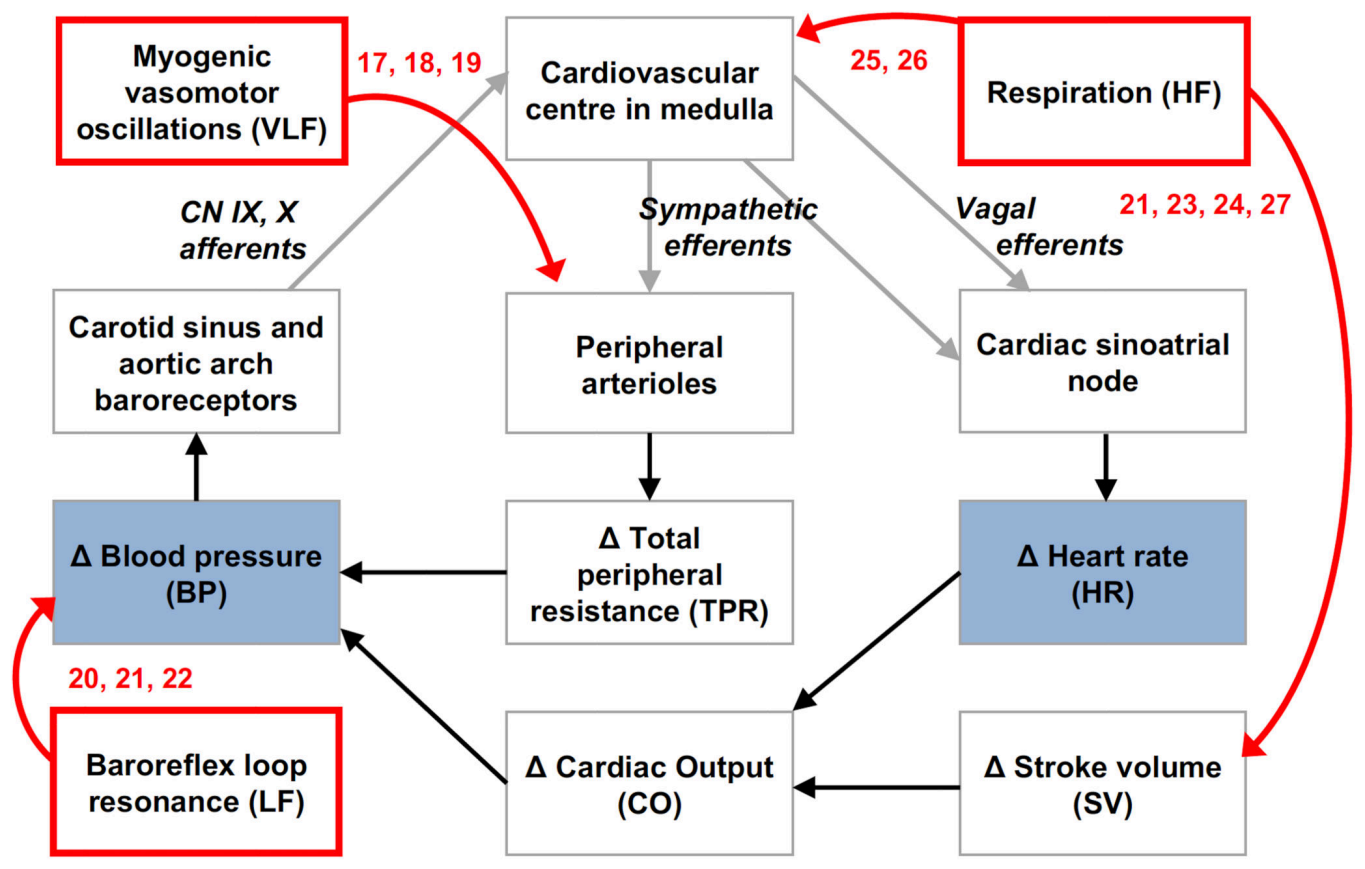
Origins of arterial blood pressure (BP) and heart rate (HR) variability. Acute changes in BP are restored via the baroreflex. A change in BP is detected by arterial baroreceptors which signal to the medulla. This triggers a compensatory change in HR, and thus CO, via reciprocal modulation of sympathetic and vagal activity to the cardiac sinoatrial node. There is also a change in sympathetic outflow to peripheral arterioles, resulting in a compensatory change in total peripheral vascular resistance (TPR). Very low frequency (VLF, red box) blood pressure variability (BPV) occurs due to myogenic responses creating a VLF oscillation in peripheral arteriolar tone and thus TPR. The VLF BPV activates the baroreflex leading to compensatory VLF HRV. Low frequency (LF) BPV and HRV originate from baroreflex loop resonance (red box). At the resonant frequency, the time delay in this negative feedback loop means the input and output are in phase, generating self-sustained oscillations. High frequency (HF) BPV and HRV correspond to Respiration (red box). The mechanical changes during respiration lead to HF BP oscillations (inspiration lowers intrathoracic pressure, leading to increased venous return, SV and therefore CO) which then activates the baroreflex to produce compensatory HR oscillations.

Alterations in blood pressure variability (BPV) and heart rate variability (HRV) are clinically relevant non-invasive biomarkers suitable for human translation. Regulatory mechanisms of arterial blood pressure homeostasis include very low, low, and high frequency (VLF, LF, HF) BPV and HRV stemming from myogenic vasomotor oscillations^17–19^, baroreflex loop resonance^20–22^, and effects of respiration^21, 23–27^ (Fig. 1). A reduction in endothelial NO is known to augment myogenic vascular responses and therefore increase VLF BPV.^19, 28–30^ Increased VLF HRV and LF BPV (baroreflex resonance) are related to enhanced sympathetic activity in the peripheral vasculature.^21–23, 31, 32^ LF HRV reflects the combined cardiac sympathetic and vagal inputs to the sinoatrial node, while HF HRV reflects purely cardiac vagal activity.^31, 33–35^ Consequently, the LF/HF ratio of HRV is an established marker of the cardiac sympatho-vagal balance. Therefore, an increase in LF HRV in normalised units (LF_nu_ =LF/(LF+HF)) indicates cardiac sympathetic dominance, while an increase in the HF HRV in normalised units (HF_nu_ =HF/(LF+HF)) indicates vagal dominance.^31, 33–35^

Animal studies in several laboratories have established that chronic fetal hypoxia increases the generation of reactive oxygen species (ROS) in the placenta, the fetal heart and vasculature, resulting in oxidative stress in the feto-placenta unit.^1, 3, 6, 7, 9, 36–40^ Previous work from our group in rat and sheep pregnancy reported that maternal treatment with the antioxidant Vitamin C reduced fetal oxidative stress and protected against the programming of systemic hypertension in adult offspring of hypoxic pregnancy.^6, 9^ However, only high doses of Vitamin C, incompatible with human treatment, proved effective.^6, 9^ These data indicated that a targeted antioxidant therapy providing a pharmacologically relevant dose could prove suitable for clinical translation. MitoQ is a mitochondria-targeted antioxidant, consisting of a quinone group covalently linked to a triphenylphosphonium cation by a 10-carbon chain.^41^ The cation drives MitoQ bioaccumulation within mitochondria, due to the negative transmembrane potential. Within the mitochondrial matrix the ubiquinone group is reduced to ubiquinol by complex II of the electron transport chain, and oxidised to ubiquinone by scavenging ROS, thereby maintaining a self-sustaining pool.^41^ Because mitochondria are the main producers of cellular ROS^42^, MitoQ is effective at low doses lower systemic doses than Vitamin C, providing a mitochondria-targeted therapy of improved clinical translation.^41^ Phase II trials have shown that MitoQ can be given safely to humans at doses which are protective against pathologies involving mitochondria-derived oxidative stress.^41^

Therefore, in the present study, we used an established rat model of hypoxic pregnancy to test the inter-related hypotheses that programmed cardiovascular dysfunction in the young adult offspring and its amelioration by maternal treatment with MitoQ can both be identified at the whole organism level through alterations in BPV and HRV, providing non-invasive biomarkers for clinical translation.

## Methods

The data that support the findings of this study are available from the corresponding author upon reasonable request.

### Ethical approval

All experiments were carried out under the UK Animals (Scientific Procedures) Act, 1986 Amendment Regulations 2012 following review by the University of Cambridge Animal Welfare Ethical Review Body and conducted in accordance with these regulations. Reporting conforms with the ARRIVE Guidelines.

### Generation of experimental groups

Wistar rats (Charles River Ltd, Margate, UK) were housed under standard conditions: 21% oxygen (O_2_), 60% humidity, 21°C, a 12-hour light 12-hour dark cycle, with free access to food (maintenance diet; Charles River Ltd) and water. Following 14 days of acclimatisation, nulliparous females were paired with fertile males (minimum 12 weeks of age). The presence of a copulatory plug was defined as day 0 of gestation. Pregnant females (dams) were housed individually under the established conditions.

**Figure 2:**
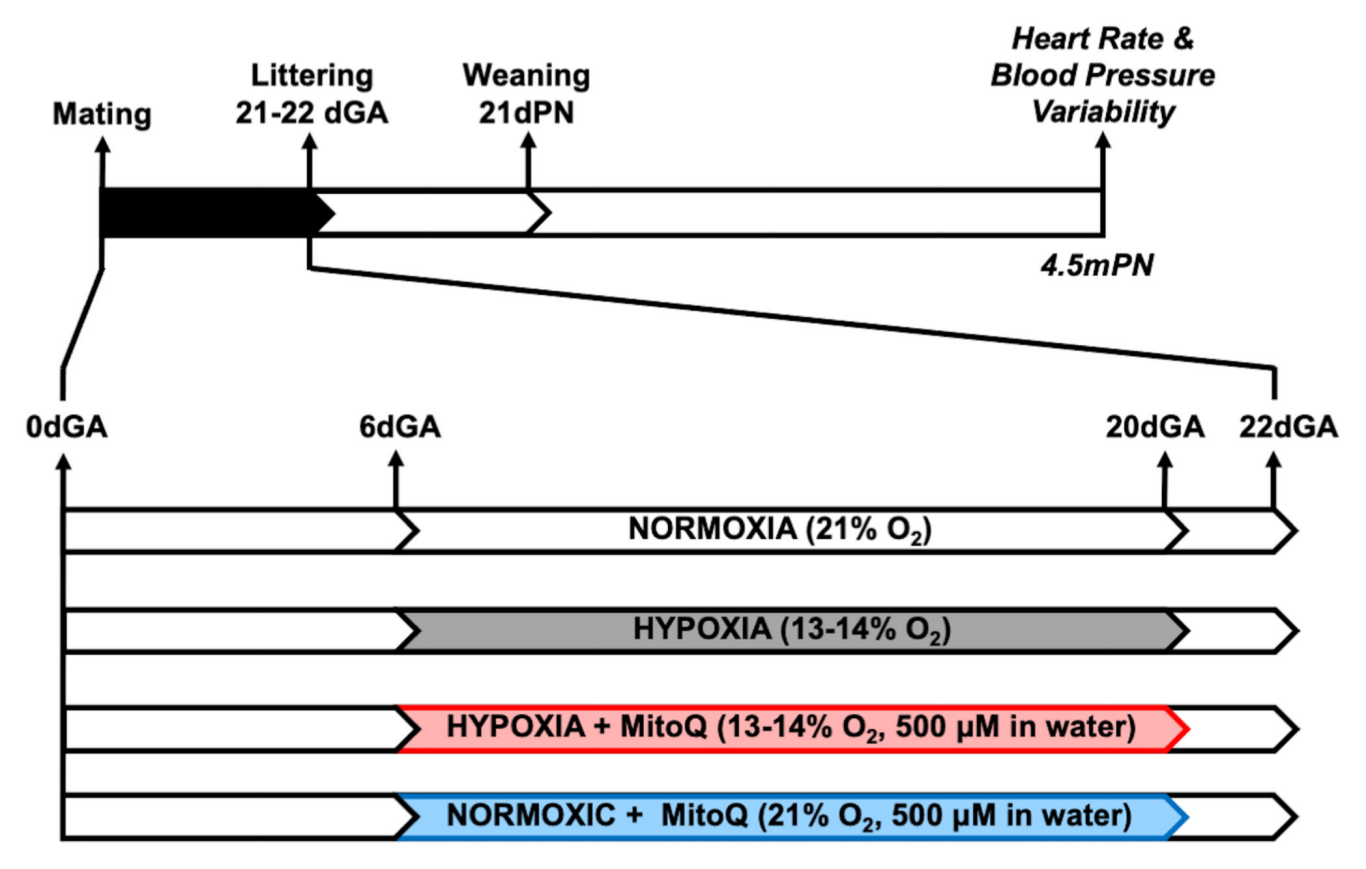
Experimental design. Days gestational age (dGA) for the induction of prenatal hypoxia, and MitoQ intervention, weaning at 21 postnatal days (dPN), and cardiovascular assessment at 4.5 postnatal months (mPN).

On day 6 of gestation, dams were randomly allocated to one of four groups: normoxia (N), hypoxia (H), hypoxia+MitoQ (HM) or normoxia+MitoQ (NM). From day 6-20 of gestation, dams in the hypoxic groups (H and HM) were placed within a chamber combining a PVC isolator and nitrogen generator. The chamber housed up to 9 rat cages in a tranquil environment.^9, 43^ By varying nitrogen inflow against constant air inflow, the O_2_ fraction was maintained at 13-14%.^1^ This simulates the reduction in oxygenation experienced at 3500m altitude.^44^ This level of hypoxia also results in a 20-30% decrease in PO_2_ in the fetal circulation ^44–46^ which corresponds to the fall in oxygenation measured by cordocentesis in human infants in pregnancy complicated by fetal growth restricted pregnancy or preeclampsia.^47, 48^ Therefore, the level of hypoxic pregnancy induced is human clinically relevant. Exposing pregnant Wistar rats to 13-14% O_2_ from day 6-20 of gestation does not reduce maternal food intake,^1, 9, 43^ allowing the effect of hypoxia to be assessed independent of changes in maternal nutrition. Maternal hypoxia was initiated on day 6, as significant pregnancy loss can be triggered if initiated prior to this time point.^9, 43^ From day 6-20 of gestation, dams in the MitoQ treatment groups (HM and NM) were provided with MitoQ at 500μM/L in their drinking water, which was made fresh every day. Previous rodent studies show that similar doses can be given safely long term, and that these doses are protective in pathological models.^1, 41^ On day 20 of pregnancy all dams were returned to normoxia and normal drinking water, and allowed to litter naturally (day 21-22). At 2 days postnatal age, pups were sexed by measurement of anogenital distance, weighed, and litters reduced to eight pups with an equal sex ratio to standardize feeding and maternal care. Offspring were maintained in normoxic conditions postnatally. All pups remained with their mothers until weaning at postnatal day 21. After weaning, rats were group-housed under standard conditions and maintained until 4.5 months of age (Fig. 2). Maternal and offspring morphometrics were collected (Table S1). No rats were euthanised for the purposes of this work. Following cardiovascular assessment, all rats were allocated to undergo further experimentation published previously,^1^ after which they were euthanised by CO_2_ inhalation and posterior cervical dislocation.

### Cardiovascular assessment *in vivo*

To control for sex and within litter variation, one male per litter was randomly assigned for cardiovascular assessment at 4.5 months of age. Rats were surgically instrumented under 2.0-2.5% isoflurane general anaesthesia. Adequate depth of anaesthesia was confirmed and monitored by the absence of corneal and limb withdrawal reflexes. The femoral artery and vein were isolated under a dissecting microscope and catheters prefilled with heparinised saline (100 U·ml^-1^ heparin in 0.9% NaCl) were introduced.^1, 43^ A customised Transonic flow probe (0.7PSL Back Exit NanoProbe, Transonic, US) was implanted around the femoral artery of the contralateral leg, and the flow probe and catheters were exteriorised at the shoulder with a dual-channel vascular access harness.^1^

Rats were acclimatised to the testing cage for 30 minutes daily for 4-5 days, and immediately prior to the testing procedures. On the fifth post-operative day, the arterial catheter was flushed and connected to a fluid-filled pressure transducer. Continuous baseline descending aortic arterial blood pressure and femoral blood flow were recorded for 30 minutes with a PowerLab 4/25 on LabChart Pro 8.0 (both AD Instruments). All recordings were made in the afternoon to control for the effect of circadian rhythm. Spontaneous movement was noted on the LabChart recording.^1^

### Blood pressure and heart rate variability analysis

Two non-overlapping 5-minute epochs from the 30-minute recording at the same time of the day were selected for BPV and HRV analysis. In this study, 5-minute recording epochs were selected in order to optimise the accuracy of VLF component analysis. Because rodents have a much higher heart rate than humans, less than 5-minute epochs of recording are acceptable.^49^ The 5-minute epochs were selected to be as close to the end of the basal recording period as possible, with those containing movement artefacts excluded. Each epoch was inspected manually, and the peak detection height adjusted until all peaks were detected.

Systolic BPV was calculated based on the variation in the peak heights in the blood pressure recording and HRV based on the variation in inter-peak interval lengths in either the blood pressure or blood flow recording (Fig S1. A,B). For BPV analysis, the values for systolic blood pressure were plotted against time, resampled at 10Hz in accordance with the Nyquist-Shannon sampling theorem,^50, 51^ and transformed into the frequency domain using the Fast Fourier Transform (FFT) algorithm (FFT size 1KHz, Hann window with 50% overlap). The DC component at 0Hz was removed. For HRV analysis, the values for inter-heartbeat interval were plotted against time and the standard deviation of inter-beat intervals (SDNN), a commonly reported time domain measure of overall HRV, was calculated. The data were then Fast Fourier Transformed using the HRV analysis module in LabChart. For both BPV and HRV, the amount of variation at each frequency was displayed as a power spectrum (Fig S1.C, D). LabChart was programmed to calculate the power in the VLF, LF and HF frequency ranges. For HRV, the LF/HF ratio, LF_nu_ (=LF/(LF+HF)) and HF_nu_ (=HF/(LF+HF)) were also calculated. The frequency boundaries were set at VLF= 0-0.2Hz, LF=0.2-0.75Hz and HF=0.75-3.0Hz based on previous studies in adult Wistar.^52^ Visual inspection of the spectra showed that each of the main peaks was confined to one frequency range, confirming that these boundaries were appropriate.

Systolic blood pressure has been used for BPV analysis in previous rodent studies.^17, 53, 54^ Although HRV is traditionally calculated based on the interval between R waves in an electrocardiogram, studies have shown that blood pressure and flow recordings provide comparable results.^55–57^ Some rats had pulsatile recordings for blood flow but not blood pressure possibly due to displacement of the catheter, so only HRV could be analysed.

### Statistical analysis

Based on previous cardiovascular studies of offspring of hypoxic pregnancy, we calculated that to detect a statistically significant difference in femoral vascular resistance in adult rats of 25%, with 95% power and a 2-tailed significance of 0.05, *n*=8 per experimental group were required. Allocation to treatment was randomised and analysis was blinded to avoid bias. All graphical and statistical analyses were carried out using GraphPad Prism 7 (GraphPad Software Inc, USA). Distribution was verified with the Shapiro-Wilk test, and statistical comparisons were made using one-way analysis of variance (ANOVA) for differences between the groups, and two-way ANOVA for the effect of hypoxia, the effect of MitoQ, and any interaction. For correlations between measures, the Pearson correlation coefficient (R^2^) was calculated. For all comparisons, significance was set at p<0.05. Data are expressed as the mean ± the standard error of the mean (SEM).

## Results

### Basal arterial blood pressure and heart rate

**Figure 3:**
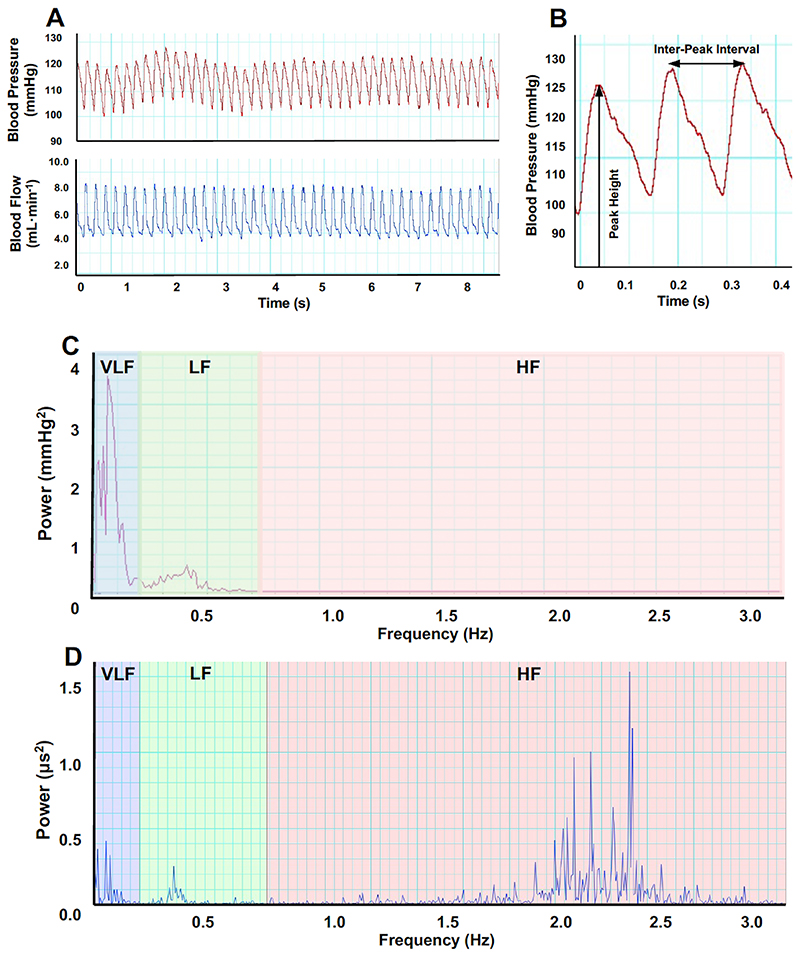
Cardiovascular recording analysis. Representative LabChart recording of continuous, pulsatile arterial blood pressure (red trace) and femoral blood flow (blue trace) from a normoxic rat (A). Expanded section of the blood pressure trace (B); the peak height (systolic blood pressure) and inter-peak interval are noted. Values for systolic blood pressure and inter-peak interval were plotted against time, and fast Fourier transformed into the frequency domain to produce BPV (C) and HRV (D) power spectra, respectively.

When the offspring were 4.5 months, mean arterial blood pressure was not significantly different (p=0.42) between the four treatment groups (Fig. 3A). Mean heart rate was also not significantly different (p=0.33) between the four treatment groups (Fig. 3B).

### Systolic blood pressure variability

**Figure 4:**
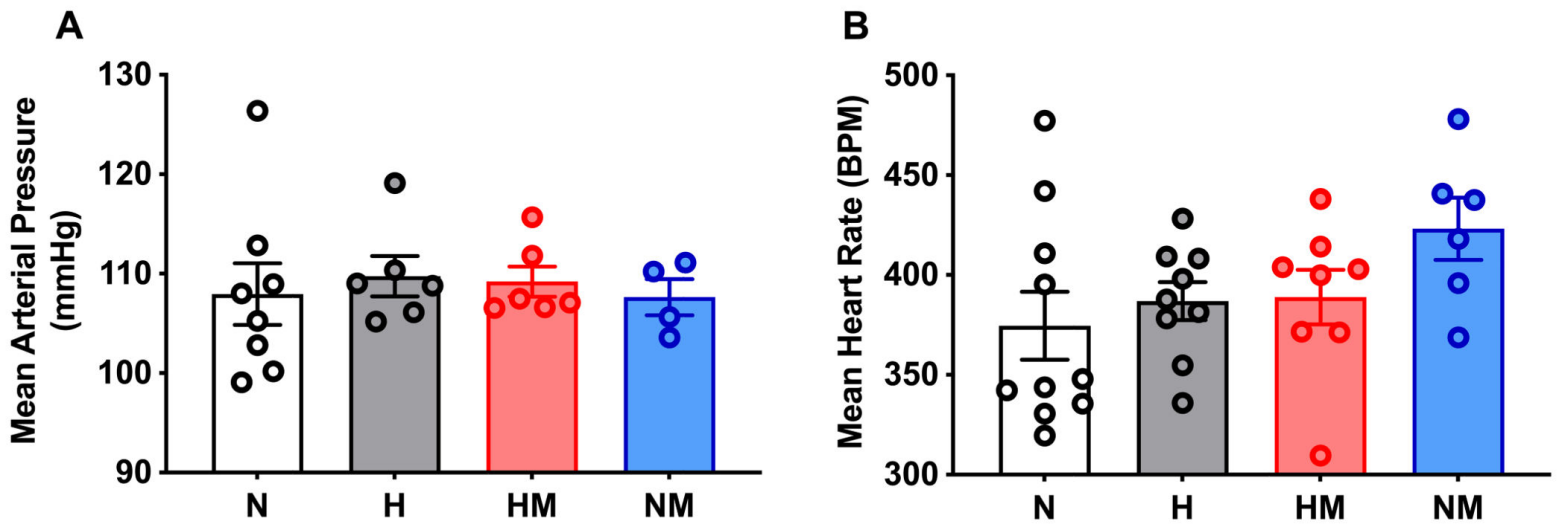
Baseline cardiovascular function. Mean arterial pressure (MAP, A) and mean heart rate (HR, B) in male offspring from normoxic (N; white, n=8 and 10, respectively), hypoxic (H; grey, n=6 and 9, respectively), hypoxic+MitoQ (HM; red, n=6 and 8, respectively), and normoxic+MitoQ (NM; blue, n=4 and 6, respectively) pregnancies. Data are mean ± SEM. Two-way ANOVA for the effect of hypoxia (*, p <0.05) and the effect of MitoQ (†, p <0.05).

Offspring of hypoxic pregnancy had increased (p=0.026) VLF BPV compared with normoxic offspring. Offspring from pregnancies treated with maternal MitoQ had decreased (p=0.049) VLF BPV compared with offspring from untreated pregnancies, and there was no interaction (p=0.37) between the effect of hypoxia and MitoQ (Fig. 4A). Offspring from hypoxic pregnancy also had increased (p=0.001) LF BPV, but here maternal MitoQ had no effect (p=0.49, Fig. 4B). HF BPV was not different amongst the experimental groups (p=0.75: N, 0.57±0.15 mmHg^2^; H, 0.98±0.48 mmHg^2^; HM, 0.77±0.24 mmHg^2^; NM, 1.0±0.58 mmHg^2^).

### Heart rate variability

**Figure 5:**
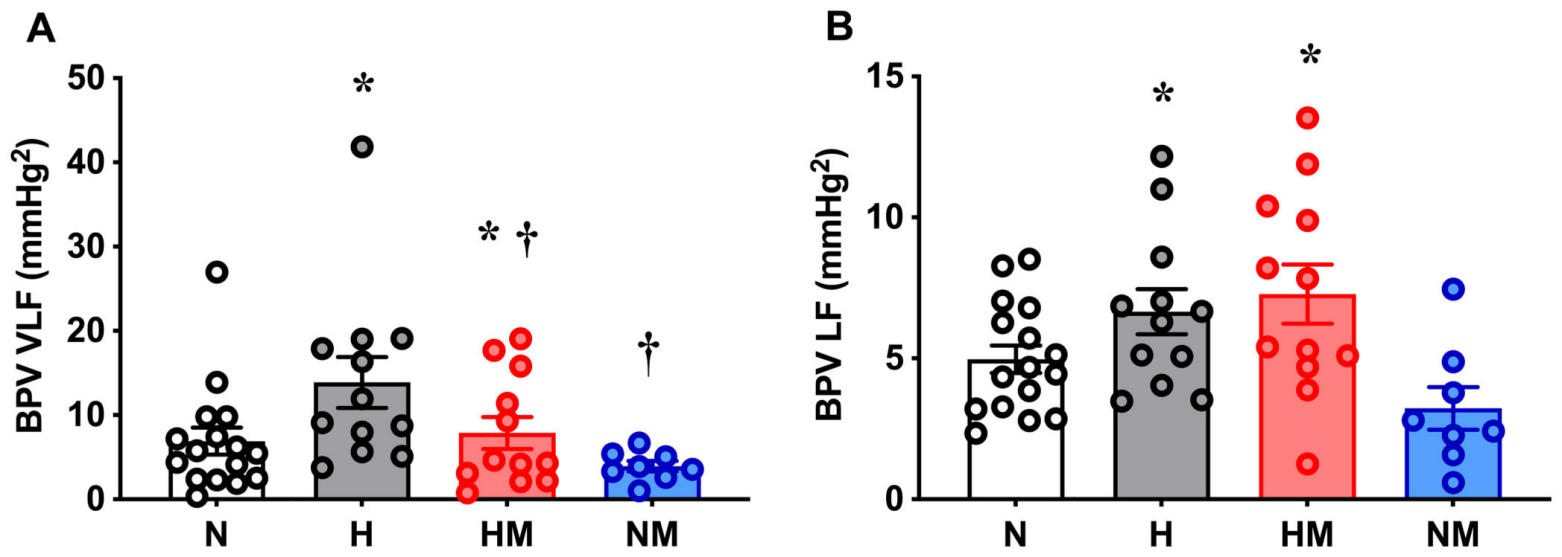
Blood pressure variability. Very low frequency (VLF, A) and low frequency (LF, B) blood pressure variability (BPV) in from male offspring from normoxic (N; white, n=8), hypoxic (H; grey, n=6), hypoxic+MitoQ (HM; red, n=6), and normoxic+MitoQ (NM; blue, n=4) pregnancies. Data are mean ± SEM. Two-way ANOVA for the effect of hypoxia (*p <0.05) and the effect of MitoQ (†p <0.05).

Offspring of hypoxic pregnancy had increased SDNN HRV (p=0.003), whilst this was decreased in offspring from pregnancies treated with maternal MitoQ (p=0.011). There was no interaction (p=0.23) between hypoxia and MitoQ (Fig. 5A). Similarly, offspring of hypoxic pregnancy had increased (p=0.008), while those from maternal MitoQ treated pregnancies showed decreased (p=0.045) VLF HRV. There was no interaction (p=0.58) between hypoxia and MitoQ (Fig. 5B). LF HRV was not significantly different amongst experimental groups (p=0.077: N, 2.43±0.88μs^2^; H,2.18±0.46μs^2^; HM, 2.99±1.08μs^2^; NM, 0.27±0.069μs^2^). HF HRV was also not different (p=0.26: N, 9.75±2.83μs^2^; H, 8.79±2.14μs^2^; HM, 10.43±3.18μs^2^; NM, 2.86±0.19μs^2^). Offspring of hypoxic pregnancy had increased (p=0.040) LF/HF ratio of HRV, while those from maternal MitoQ treatment showed no effect (p=0.26, Fig. 5C). Offspring of hypoxic pregnancy also had increased (p=0.036) LF_nu_ HRV and decreased (p=0.034) HF_nu_ HRV, and again those from maternal MitoQ treatment showed no effect (p=0.18, Fig. 5D).

### Correlations

**Figure 6:**
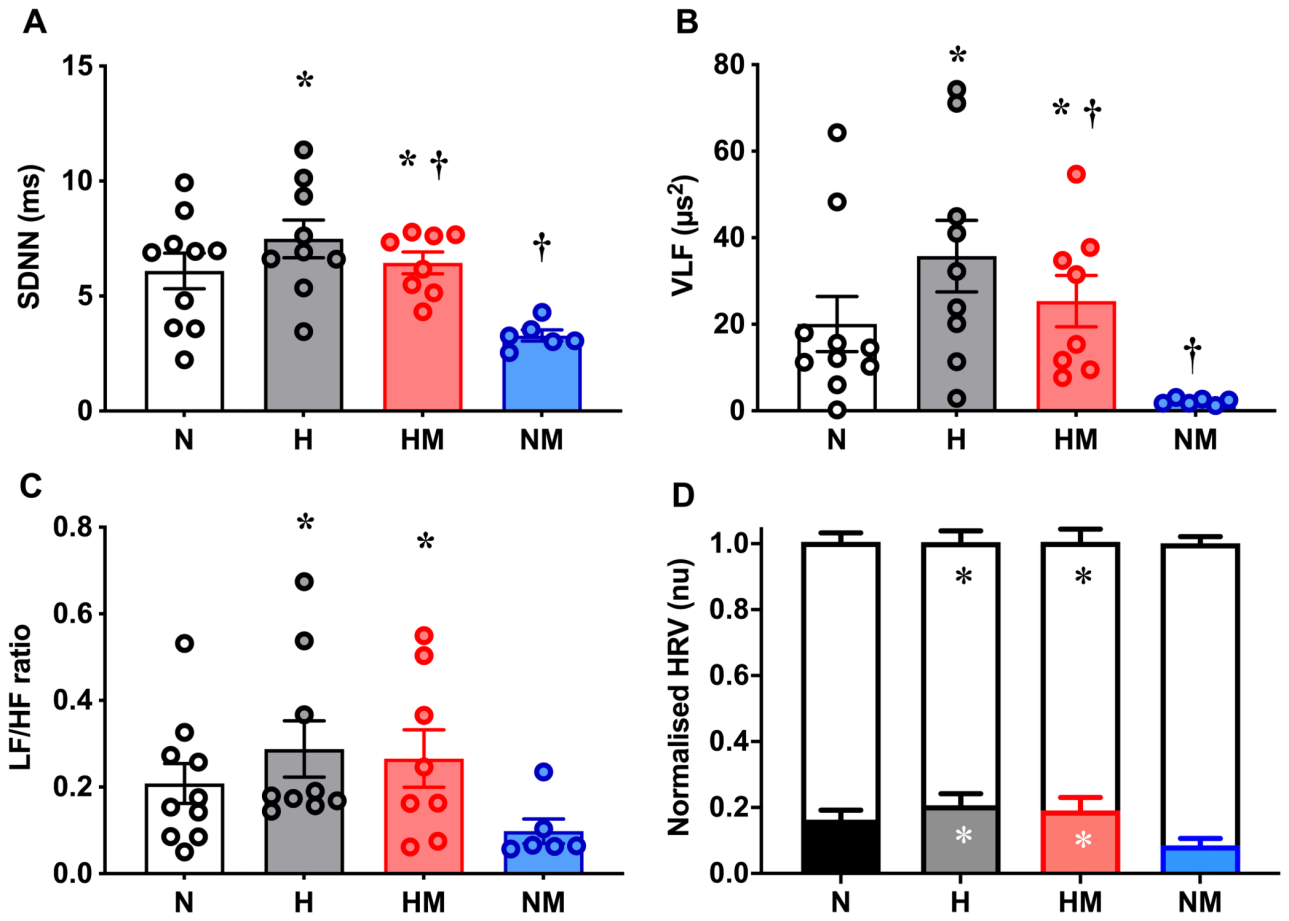
Heart rate variability. The standard deviation of the inter-beat intervals (SDNN, A), very low frequency (VLF) heart rate variability (HRV, B), low frequency/ high frequency ratio (LF/HF, C) in male offspring from normoxic (N; white, n=10), hypoxic (H; grey, n=9), hypoxic+MitoQ (HM; red, n=8), and normoxic+MitoQ (NM; blue, n=6) pregnancies, and normalised LF (black bars) and HF (white bars) HRV (D). Data are mean ± SEM. Two-way ANOVA for the effect of hypoxia (*, p <0.05) and the effect of MitoQ (†, p <0.05).

Data from all four groups showed significant positive correlations between VLF BPV and VLF HRV (Fig. 6A), and between the LF/HF ratio of HRV and LF_nu_ HRV (figure S1).

## Discussion

Consistent with our hypothesis, we show that male young adult rat offspring of hypoxic pregnancy, which we have previously reported to show abnormal cardiovascular function,^1, 5, 8, 43^ display biomarkers of impaired endothelial NO-dependent vasodilatation (increased VLF BPV and VLF HRV), as well as vascular and cardiac sympathetic hyper-reactivity (increased LF BPV and LF/HF ratio HRV, respectively), prior to the development of overt cardiovascular disease. Importantly, these biomarkers of cardiovascular dysfunction can be identified non-invasively in humans. Maternal MitoQ therapy was protective against indices associated with impaired endothelial function but had no effect on vascular or cardiac sympathetic hyper-reactivity in adult offspring of hypoxic pregnancy, highlighting the divergent programming mechanisms involved.

Alterations in BPV and HRV are clinically translatable biomarkers that can be collected from patients during routine analysis, such as with a beat-to-beat finger blood pressure monitor and electrocardiogram.^58^ Importantly, the relatively young offspring in this study, at an age which corresponds to late adolescence in humans,^59^ were not hypertensive or tachycardic and did not display symptoms of cardiovascular disease. In humans, the programmed cardiovascular changes indicated by these biomarkers precede the development of overt cardiovascular pathology. Both impaired NO-dependent vasodilatation^16^ and increased vascular sympathetic activity^14^ are implicated in the pathogenesis of hypertension. Additionally, a sustained increase in cardiac sympathetic activity stimulates cardiomyocyte apoptosis and β-adrenergic desensitisation, which contribute to the pathology of heart failure.^15^ We know offspring of hypoxic pregnancy are at increased risk of these diseases in later life.^3^ Therefore, BPV and HRV could enable earlier detection of programmed cardiovascular dysfunction and intervention.

The reasoning behind using alterations in BPV and HRV as biomarkers of cardiovascular dysfunction is underpinned by the physiology of blood pressure homeostasis. A reduction in endothelial NO-dependent vasodilatation is known to increase VLF BPV by reducing NO-mediated buffering of VLF vascular myogenic contractions.^19, 28–30^ VLF HRV is proposed to be a compensatory baroreflex response to VLF BP oscillations.^23^ Accordingly, in the present study, we found VLF BPV and HRV to be positively correlated (R^2^=0.58). Baroreflex loop resonance generates a LF oscillation in sympathetic outflow to the peripheral vasculature, resulting in LF vasoconstriction.^20, 22, 60^ As endothelial mediators cannot act fast enough to buffer vasoconstriction at this frequency, LF BPV is an established biomarker of vascular sympathetic activity.^21, 22, 31, 32^ LF HRV also corresponds to the baroreflex resonant frequency and represents combined sympathetic and parasympathetic cardiac modulation, while HF HRV, coupled with the respiratory frequency, represents purely the faster acting parasympathetic modulation.^49^ Therefore, the LF/HF ratio of HRV and LF_nu_ are established measures of cardiac sympathovagal balance.^31, 33–35^ In the present study, the robust correlation (R^2^=0.99) between the LF/HF ratio and LF_nu_ HRV, demonstrates their equivalence.

Additional data in the present study show that maternal MitoQ treatment in hypoxic pregnancy protected against the programming of indices associated with impaired endothelial NO-dependent vasodilatation, suggesting that this programming is mediated developmentally by mitochondria-derived oxidative stress or redox signaling. Importantly, the protection afforded by MitoQ treatment in hypoxic pregnancy could also be identified through non-invasive analysis of VLF BPV and HRV. One proposed mechanism underlying the programming of cardiovascular dysfunction in offspring of hypoxic pregnancy is that fetal hypoxia results in increased mitochondrial production of the ROS superoxide (O_2_•^−^), which rapidly reacts with endothelial-derived NO, reducing its bioavailability.^3, 61^ By scavenging excess mitochondria-derived O_2_•^−^ production, or by decreasing its production, MitoQ can restore NO bioavailability and thereby endothelial function. This is supported by work in hypoxic sheep pregnancy, where maternal MitoQ treatment has been found to protect against the programming of hypertension in adulthood by enhancing NO signalling in the peripheral vasculature.^7^ It is also consistent with work describing that hypoxic incubation of chicken embyos can enhance mitochondria-derived ROS production and that this is prevented by MitoQ treatment.^7^ It is also consistent with work in adult spontaneously hypertensive rats, which showed that MitoQ increases NO-bioavailability and improves endothelial function.^62^ The protective effects of MitoQ on the developing cardiovascular system may be direct and/or secondary to beneficial effects at the level of the placenta. Using a mitochondria-targeted mass spectrometry probe, we have previously reported that incubation of chicken embryos under hypoxic conditions increases the generation of mitochondria-derived ROS.^7^ The same study showed that treatment of hypoxic embryos with MitoQ normalises mitochondria-derived ROS generation, confirming a direct protective effect of MitoQ on the embryonic cardiovascular system. Similarly, we and others have reported that maternal treatment with both authentic MitoQ and nanoparticle bound MitoQ in hypoxic pregnancy reduces oxidative stress and has protective effects on the maternal side of the placenta.^37, 38, 40, 63^

Conversely, the present study show that maternal MitoQ therapy had no significant effect on indices associated with either vascular (LF BPV) or cardiac (LF/HF ratio and LF_nu_ HRV) sympathetic hyper-reactivity. These findings suggest that the programming of sympathetic hyper-reactivity in offspring of hypoxic pregnancy is mediated via mechanisms independent of mitochondria-derived oxidative stress. Fetal hypoxia is known to activate a carotid chemoreflex, which increases sympathetic outflow, mediating vasoconstriction in the peripheral vasculature.^8, 64^ This effect is part of the fetal brain-sparing response that shunts blood flow away from less essential vascular beds towards the fetal brain.^8, 64^ Persistent chemoreflex activation has been shown to lead to chemoreflex sensitisation.^65^ Additionally, there is evidence that chronic developmental sympathetic stimulation can lead to upregulation of adrenoceptors and a sustained increase in tissue sensitivity.^66^ Studies in chicken embryos have reported that developmental hypoxia programmes sympathetic hyperinnervation of the peripheral vasculature that persists into adulthood; the proposed mechanism involves activation of hypoxia inducible factor.^12, 67^ Hypoxic pregnancy in rats leads to increased femoral vasoconstrictor responses to sympathetic agonists in new born pups^13^ as well as increased muscle sympathetic nerve activity and sympathetic hyperinnervation in adult offspring.^68^ Similarly, chronic hypoxia in ovine pregnancy enhances femoral vasoconstrictor responses to the α-adrenergic agonist phenylephrine in the fetus and programmes femoral vasoconstrictor hyper-reactivity to sympathetic agonists in the adult offspring.^6^ Combined, these data suggest that chronic hypoxia programmes cardiovascular dysfunction in the adult offspring via multiple mechanisms, including mitochondria-derived oxidative stress and hyper-reactivity of the sympathetic nervous system. Therefore, maternal treatment with MitoQ in hypoxic pregnancy protected against cardiovascular symptoms triggered by mitochondria-derived oxidative stress but not via enhanced sympathetic activation. Importantly, this can be identified by differential diagnoses of the non-invasive biomarkers.

These data are of clinical relevance as they highlight that pharmacological targeting of one oxidative stress pathway may be insufficient to protect offspring from cardiovascular dysfunction programmed developmentally by adverse intrauterine conditions in human complicated pregnancy. These data also highlight that non-invasive BPV and HRV monitoring in young adult offspring of complicated pregnancy can both identify clinically relevant indicators of cardiovascular dysfunction, and differentiate clinical indicators mediated via oxidative stress or sympathetic hyper-reactivity. Therefore, non-invasive differential diagnosis could refine early intervention with mechanism-targeted therapies in young adult offspring, prior to the establishment of over cardiovascular disease.

Previous studies of developmental programming in rodent models have identified sex differences in outcomes in adult offfspring^69^. A limitation of this study is that by investigating male but not female offspring, sex differences were controlled for, but not addressed. An important advantage of BPV and HRV analyses presented in this study is that these measures can be conducted repeatedly across time. Therefore, follow-up work should determine longitudinal changes in BPV and HRV function over time with ageing, in both male and female offspring.

In summary, using an established rat model of hypoxic pregnancy, this study shows that known components of programmed cardiovascular dysfunction can be identified *in vivo* in asymptomatic male adult offspring using alterations in BPV and HRV biomarkers. Our findings also provide evidence that maternal treatment with the mitochondria-targeted antioxidant MitoQ in hypoxic pregnancy protects against the programming of indices associated with reduced NO-dependent vasodilatation, but not vascular or cardiac sympathetic hyper-reactivity. This suggests that mitochondria-derived oxidative stress is one of multiple mechanisms mediating cardiovascular programming by chronic fetal hypoxia. Clinically translatable findings include the use of BPV and HRV analysis for early identification of programmed cardiovascular dysfunction in human offspring of hypoxic pregnancy, as well as for diagnosis of effective intervention.

## Perspectives

Humans exposed to adverse conditions *in utero* have an increased cardiovascular risk in later life. Chronic fetal hypoxia is one of the most common adverse conditions in complicated pregnancy, and it is known to programme endothelial dysfunction and sympathetic-hyperreactivity in preclinical animal models. Here, we report in male rats that corresponding alterations in blood pressure (BPV) and heart rate (HRV) variability can be detected *in vivo* in young adult offspring of hypoxic pregnancy, prior to the development of overt heart disease. Therefore, BPV and HRV analysis could be useful non-invasive biomarkers for early identification of subclinical programmed cardiovascular dysfunction in humans. We also show that maternal treatment with the mitochondria-targeted MitoQ in hypoxic pregnancy prevents the programming of indices associated with endothelial dysfunction, but not of sympathetic hyper-reactivity, in the adult offspring. Therefore, programmed cardiovascular disease and underlying mechanisms can be differentially diagnosed using biomarkers that can be measured non-invasively in the human clinical setting. This perspective offers the improved clinical diagnosis and targeted treatment of offspring affected by cardiovascular dysfunction which has been programmed by their own adverse intrauterine environment.

## Supplementary Material

Online Supplement

## Novelty and Significance

**What is New?**

- Here we show in rats that cardiovascular problems in male adult offspring programmed by hypoxic pregnancy can be diagnosed *in vivo* simply by analysis of blood pressure and heart rate variability, identifying non-invasive biomarkers that could be translated to the human clinical setting.

**What is Relevant?**

- Pregnancy affected by chronic fetal hypoxia, one of the most common pregnancy complications in humans, increases the risk of cardiovascular dysfunction in later life, including hypertension.

**Summary**

- We have identified non-invasive clinical biomarkers to pick-up asymptomatic cardiovascular dysfunction in male adult offspring programmed by developmental hypoxia
