# Supplementary material for: Non-Invasive Biomarkers for Cardiovascular Dysfunction Programmed in Male Offspring of Adverse Pregnancy
Source: Hypertension. Author manuscript; Available in PMC 2022 May 4. (PMC8577293; doi:10.1161/HYPERTENSIONAHA.121.17926)
Supplement: Online Supplement [file EMS135436-supplement-Online_Supplement.docx]

NON-INVASIVE BIOMARKERS FOR CARDIOVASCULAR DYSFUNCTION PROGRAMMED IN MALE OFFSPRING OF ADVERSE PREGNANCY

^*^Rama Lakshman^1^, *Ana-Mishel Spiroski^1,3^, Lauren B. McIver^1^, Michael P. Murphy^2,3,4^, and Dino A. Giussani^1,3,4,5^

^1^ *Department of Physiology, Development and Neuroscience,*

*University of Cambridge,* UK

^2^ *MRC Mitochondria Biology Unit, University of Cambridge, UK*

^3^ *Cambridge BHF Centre of Research Excellence, University of Cambridge, UK*

^4^ *Department of Medicine, University of Cambridge,* UK

^5^ *Cambridge Strategic Research Initiative in Reproduction, Cambridge, UK*

*Authors contributed equally to this work

Part of this work was awarded the Pfizer President’s Presenter’s Award at the 65^th^ Annual International Meeting of the Society for Reproductive Investigation, San Diego, California, USA.

**Short Title**: Non-invasive biomarkers for programmed heart disease

**Corresponding Author**: Professor Dino A. Giussani PhD ScD FRCOG

Department of Physiology, Development and Neuroscience

University of Cambridge

Downing Street, Cambridge

CB2 3EG

UK

Email: dag26@cam.ac.uk

Tel: +44 (0)1223 333894

| **table S1. Maternal and offspring characteristics.** | | | | | | | | |  |
| --- | --- | --- | --- | --- | --- | --- | --- | --- | --- |
|  | Experimental Group | | | |  | Significance  (treatment effect) | | |  |
| **Variable** | N | H | HM | NM |  | Hypoxia | MitoQ | Interaction |  |
| ***Dams, 7-20 dGA*** | *n*=10 | *n*=10 | *n*=6 | *n*=8 |  |  |  |  |  |
| **Food intake (g·kg·d^-1^)** | 79±2 | 70±3 | 73±3 | 75±3 |  | ns | ns | ns |  |
| ***Litter characteristics, 2 dPN*** | *n*=10 | *n*=9 | *n*=7 | *n*=9 |  |  |  |  |  |
| **Litter size** | 14±1 | 9±1 | 11±1 | 12±1 |  | <0.05 | ns | ns |  |
| **Sex ratio (male:female)** | 1.1±0.2 | 0.9±0.2 | 1.5±0.2 | 0.7±0.2 |  | ns | ns | <0.05 |  |
| ***Postnatal offspring*** | *n*=10 | *n*=9 | *n*=8 | *n*=9 |  |  |  |  |  |
| **2 dPN weight (g)** | 7.7±0.4 | 6.9±0.4 | 7.4±0.5 | 7.9±0.4 |  | ns | ns | ns |  |
| **16 wPN weight (g)** | 530±13 | 478±13 | 484±16 | 496±14 |  | <0.05 | ns | ns |  |
| Maternal, litter and offspring measures were collected as indicated in normoxic (N), hypoxic (H), hypoxic MitoQ (HM) and normoxic MitoQ (NM) pregnancies. Data are means ± SEM. Statistical differences are (p<0.05): main effect of hypoxia; main effect of MitoQ, and hypoxia x MitoQ interaction (Two-Way ANOVA with Tukey’s post-hoc comparison). Days gestational age (dGA), postnatal days (dPN), postnatal weeks (wPN). | | | | | | | | |  |

**figure S1. Variability parameter correlations.** The correlation between very low frequency (VLF) blood pressure variability (BPV) and heart rate variability (HRV; A, n=24), and normalised low frequency (LF) and low frequency/high frequency (LF/HF) ratio of HRV (B, n=33) in all experimental animals. The Pearson correlation coefficient (R^2^) was used.

.
